# Process Evaluation of the SImplification of Medications Prescribed to Long-tErm Care Residents (SIMPLER) Cluster Randomized Controlled Trial: A Mixed Methods Study

**DOI:** 10.3390/ijerph18115778

**Published:** 2021-05-27

**Authors:** Janet K. Sluggett, Georgina A. Hughes, Choon Ean Ooi, Esa Y. H. Chen, Megan Corlis, Michelle E. Hogan, Tessa Caporale, Jan Van Emden, J. Simon Bell

**Affiliations:** 1 UniSA Allied Health and Human Performance, University of South Australia, Adelaide, SA 5001, Australia; megan.corlis@unisa.edu.au; 2 Centre for Medicine Use and Safety, Faculty of Pharmacy and Pharmaceutical Sciences, Monash University, Parkville, VIC 3052, Australia; choonean.ooi@monash.edu (C.E.O.); esa.chen@monash.edu (E.Y.H.C.); simon.bell2@monash.edu (J.S.B.); 3 NHMRC Cognitive Decline Partnership Centre, Hornsby Ku-ring-gai Hospital, Hornsby, NSW 2077, Australia; Jan.VanEmden@unisa.edu.au; 4 UniSA Clinical and Health Sciences, University of South Australia, Adelaide, SA 5001, Australia; georgina.hughes@mymail.unisa.edu.au; 5 Helping Hand Aged Care, North Adelaide, SA 5006, Australia; mehogan@helpinghand.org.au (M.E.H.); tcaporale@helpinghand.org.au (T.C.); 6 Department of Epidemiology and Preventive Medicine, Monash University, Melbourne, VIC 3004, Australia

**Keywords:** process assessment, health care, aged, nursing homes, long-term care, residential facilities, medication therapy management, medication administration, medication systems, qualitative research, randomized controlled trial, Australia

## Abstract

Complex medication regimens are highly prevalent, burdensome for residents and staff, and associated with poor health outcomes in residential aged care facilities (RACFs). The SIMPLER study was a non-blinded, matched-pair, cluster randomized controlled trial in eight Australian RACFs that investigated the one-off application of a structured 5-step implicit process to simplify medication regimens. The aim of this study was to explore the processes underpinning study implementation and uptake of the medication simplification intervention. A mixed methods process evaluation with an explanatory design was undertaken in parallel with the main outcome evaluation of the SIMPLER study and was guided by an established 8-domain framework. The qualitative component included a document analysis and semi-structured interviews with 25 stakeholders (residents, family, research nurses, pharmacists, RACF staff, and a general medical practitioner). Interviews were transcribed verbatim and reflexively thematically content analyzed. Descriptive statistics were used to summarize quantitative data extracted from key research documents. The SIMPLER recruitment rates at the eight RACFs ranged from 18.9% to 48.6% of eligible residents (38.4% overall). Participation decisions were influenced by altruism, opinions of trusted persons, willingness to change a medication regimen, and third-party hesitation regarding potential resident distress. Intervention delivery was generally consistent with the study protocol. Stakeholders perceived regimen simplification was beneficial and low risk if the simplification recommendations were individualized. Implementation of the simplification recommendations varied between the four intervention RACFs, with simplification implemented at 4-month follow-up for between 25% and 86% of residents for whom simplification was possible. Good working relationships between stakeholders and new remunerated models of medication management were perceived facilitators to wider implementation. In conclusion, the one-off implicit medication simplification intervention was feasible and generally delivered according to the protocol to a representative sample of residents. Despite variable implementation, recommendations to simplify complex regimens were valued by stakeholders, who also supported wider implementation of medication simplification in RACFs.

## 1. Introduction

Medication regimen complexity is prevalent in residential aged care facilities (RACFs). Simplifying medication regimens for residents could lead to benefits such as improved resident wellbeing, more staff time for other care activities, safer medication administration practices and greater resident involvement in medication-related decision making [1,2,3]. Trialing medication simplification activities in RACFs is important due to associations between complex medication regimens and medication administration errors, unplanned hospitalizations and mortality in older people [4,5].

The SImplification of Medications Prescribed to Long-tErm care Residents (SIMPLER) study was a non-blinded, matched-pair, cluster randomized controlled trial (RCT) of a single multidisciplinary intervention targeting complex medication regimens [1,3,6,7,8]. SIMPLER was the first prospective study to investigate the benefits and harms of medication regimen simplification in RACFs. Medication simplification involves consolidating administration times (e.g., by administering medications at the same time, switching to combination products or long-acting formulations) without changing the therapeutic intent of the medication regimen [1,3,9]. The SIMPLER study protocol and findings have been described previously [1,3,6,7,8]. In brief, residents at four RACFs were randomized to receive usual care, while those in four matched RACFs received a medication simplification intervention delivered by an experienced clinical pharmacist using the 5-item Medication Regimen Simplification Guide for Residential Aged CarE (MRS GRACE) [9]. This implicit, validated tool was designed to guide the process of medication simplification in RACFs. It encourages health professionals to consider resident-related factors, regulatory or safety imperatives, potential for interactions involving medications, and alternative medication formulations that are available when determining whether a medication regimen can be simplified [9]. It also prompts the health professional to consider potential unintended consequences of simplification such as increased medication costs [9]. Medication simplification was possible for 65% of residents in the intervention arm. The mean number of medication administration times each day for regular medications was reduced in the intervention group versus the comparison group (−0.36, 95% confidence interval (CI) −0.63 to −0.09) at 4-months [3] and was sustained at 12-month follow-up [6] (Supplementary Table S1).

Process evaluations, often undertaken to understand why interventions are effective [10] and facilitate translation into practice [11], are particularly important for medication interventions in RACFs. Conducting empirical research in RACFs can present numerous challenges [12,13,14,15]. The medication management cycle in RACFs is complex and involves multiple stakeholders, and culture and processes can differ across RACFs, even those within the same organization. In Australian RACFs, which are similar to nursing homes and long-term care facilities, physicians and pharmacists are usually employed externally to the RACF, and staff administering medications may have good resident knowledge but might not be able to identify all simplification opportunities [16]. Residents of Australian RACFs are increasingly older and frailer, with complex multimorbidity and polypharmacy. Previous process evaluations of cluster RCTs of healthcare interventions suggest multiple facility, resident, staff, health professional and investigator-related factors can impact on recruitment and intervention delivery in RACFs [12,14,17,18]. These include organizational leadership, familiarity with research, trust, health literacy, attrition, resource availability and prioritization of other care activities, quality of information provided by study investigators, interprofessional communication, complexity of the intervention and mode of intervention delivery. It is therefore imperative to understand processes underpinning interventions to optimize medication management in RACFs. The aim of this process evaluation was to explore the processes underpinning the implementation and uptake of the medication simplification intervention in the SIMPLER study.

## 2. Materials and Methods

### 2.1. Study Design and Setting

A mixed methods process evaluation with an explanatory design comprising document analysis and semi-structured interviews was undertaken. The study was conducted in accordance with the 8-domain Grant et al. framework for process evaluations of cluster RCTs of health interventions [11]. This framework enables examination of processes at both the individual and cluster level and has been utilized to evaluate cluster RCTs to optimize medication use in community settings [19,20]. The data sources examined for each domain are outlined in Supplementary Table S2. The ‘intervention maintenance’ domain [11] was adapted to focus on future implementation because SIMPLER tested a one-off intervention.

**Figure 1:**
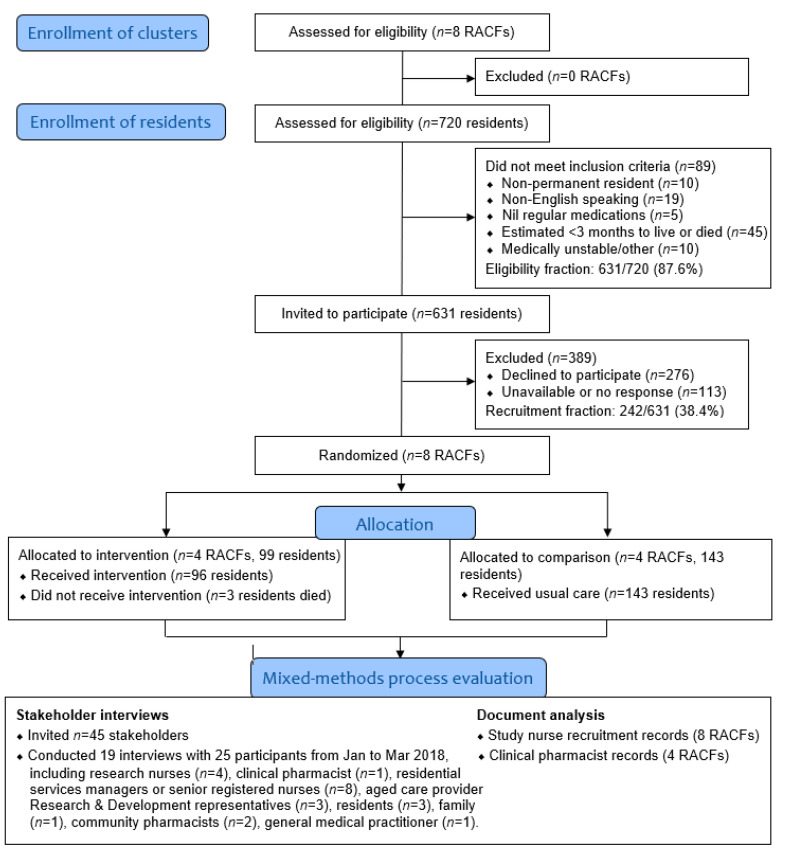
Study flow diagram describing eligibility and recruitment for the SIMPLER study at the cluster and individual resident levels.

Overall, 242 residents from eight South Australian RACFs operated by the same not-for-profit organization participated in SIMPLER (Supplementary Table S1, Figure 1). The process evaluation was conducted post-intervention, immediately following the completion of the data collection for 4-months follow-up of the SIMPLER study, but prior to data analysis [3]. This maximized participant recall and ensured researchers and participants were not influenced by knowing the 4-month outcomes.

### 2.2. Document Analysis

One researcher (G.A.H.) undertook the document analysis [21]. Field notes and records, including research nurse recruitment logs, clinical pharmacist logs and intervention reports, meeting minutes, presentations, quality improvement reports, and emails were examined (Supplementary Table S2). Quantitative and qualitative data were extracted. 

### 2.3. Semi-Structured Interviews

Semi-structured interviews were conducted with study participants, family members who provided third-party consent for residents to participate in SIMPLER, aged care provider research and development team members, research nurses, the clinical pharmacist who delivered the intervention, RACF managers and staff, GPs, and community pharmacists. The research team reviewed trial documents and asked the research nurses and clinical pharmacist to identify potential participants, who were then randomly selected and consent to participate was obtained. Individuals from comparison and intervention RACFs were included.

Overall, 25 of the 45 individuals approached provided consent to participate and were interviewed (Figure 1). All interviews were conducted by two researchers (J.K.S. and G.A.H.) using an interview guide informed by Grant et al. [11]. The guide was pilot tested for face validity with two health professionals and a consumer, and further refined thereafter. The guide was subsequently utilized for a pilot and feasibility study examining medication simplification in community-dwelling people [22]. Twenty-two participants were interviewed face-to-face and three were interviewed via videoconference (mean duration 35.9 (±14.9) min). Interviews were conducted until data saturation was achieved. Interviews were audio-recorded and transcribed verbatim by an external transcription service. No repeat interviews were conducted.

### 2.4. Qualitative Data Analysis

The qualitative component was informed by a phenomenological approach. Qualitative data analysis was conducted using an iterative process so that findings informed subsequent data generation and exploration. Three researchers (J.K.S., G.A.H. and C.E.O.) independently read all interview transcripts and information collected during the document analysis, and undertook reflexive thematic analysis using the procedures outlined by Braun and Clarke [23,24]. This involved repeated reading and familiarization, and generation of initial codes independently using a deductive approach that considered existing topic knowledge (e.g., aligning to the Grant et al. framework [11], SIMPLER study context, existing medication management interventions in RACFs, and the behavior changes theories underpinning Australia’s National Strategy for Quality Use of Medicines [25]). For each framework domain, similar codes were collated into potential themes, which were then reviewed and defined. The codes were then reviewed by all three investigators, who also identified interview quotes that illustrated key themes after writing the results.

### 2.5. Quantiative Data Analysis

Quantitative data collected during the document analysis were summarized using descriptive statistics, determined using SAS version 9.4 (SAS Institute Inc., Cary, NC, USA).

## 3. Results

### 3.1. Recruitment of RACFs (Clusters)

All eight RACFs approached agreed to be involved. In two RACFs the recruitment start date was adjusted to accommodate other quality improvement activities. Qualitative evaluation showed RACF participation was facilitated by senior staff appreciation for the importance of the study objective, opportunities to improve workflow, research team flexibility with respect to recruitment commencement, and the interdisciplinary nature of the project.

“*There was no reason not to jump on board… it made perfect sense. And we’d also get feedback from the staff, you know, that they’re very busy so anything that might reduce or improve the workflow for them was of interest.*”

[Residential Services Manager 1]

RACF recruitment was also facilitated by the involvement of an experienced clinical pharmacist in intervention delivery and recognition that new staff knowledge may persist post-intervention. Recruitment barriers included concern about the amount of staff time required on top of existing quality improvement activities.

### 3.2. Recruitment and Reach among Residents

SIMPLER study participants were recruited by research nurses employed by the aged care provider organization [1]. The nurses were trained over two days and supported by a researcher (JKS) embedded within the aged care provider organization. Box 1 summarizes the SIMPLER study promotion and recruitment strategies, which were adapted on a site-by-site basis to accommodate RACF preferences (no RACFs utilized all strategies). Research nurses suggested that in some RACFs, promotional materials could have been implemented earlier to aid recruitment. The research nurses aimed to finish the bulk of the recruitment activities at an RACF before commencing at the next RACF. Four research nurses undertook recruitment activities over a six and a half-month period, with a median of 20 days (interquartile range (IQR) 14–28) between the recruitment start dates at each RACF.

Box 1 **Strategies used to support recruitment of RACFs (clusters) and residents to participate in the SIMPLER study.**

- Regular face-to-face meetings with senior research and development staff within the aged care provider organization, senior staff at each facility and the organization’s Client Safety Quality Unit
- Researcher embedded within the residential aged care provider organization (2 days per week) regularly liaised with senior staff at the RACFs
- Participation in face-to-face RACF medication advisory committee meetings, senior nursing handover meetings, RACF staff meetings, Resident meetings, and Consumer and Carer Reference Group meetings
- In one RACF, the research team delivered a medication-related continuing education session to RACF staff that was followed by SIMPLER study promotional information
- Posters in staff meeting rooms and in public areas of the RACF, written information in emails and newsletters to staff, residents, and family members
- Written letters, flyers, emails and/or telephone calls to family members, local community pharmacies and GPs

Research nurses recruited residents in-person where a senior registered nurse (RN) advised the resident was able to self-consent. The research nurses visited residents in their rooms or other private areas to introduce themselves and SIMPLER, and provide an information sheet, and then aimed to return within 7-days to discuss potential participation. On occasion, the research nurse consulted a third party despite the resident being flagged as able to self-consent. Third parties were mailed information and consent forms with a reply-paid envelope and telephoned. Return of the signed consent form via post or email was required for study enrolment. A maximum of three attempts were made to contact third parties; some were contacted more often when the person expressed an interest in potentially providing consent. Some third parties spoke with the resident before providing informed consent or declining to participate.

Stakeholders reported the recruitment process was time consuming. Multiple recruitment discussions were undertaken by research nurses, with residents and third parties contacted on a median of two occasions (IQR 2–3). Overall, 631 residents met the inclusion criteria and were approached to participate, with 78 of the 239 residents who could self-consent providing informed consent to participate (32.6%) (Figure 1). Third-party consent was obtained for 164 of the remaining 392 residents (41.8%). 

Recruitment rates at the 8 RACFs ranged from 18.9% to 48.6% of eligible residents (38.4% overall). The final sample of 242 residents exceeded the minimum of 194 residents estimated by the initial SIMPLER sample size calculation [1]. Participants were similar to all residents of the eight RACFs and the wider Australian RACF population in terms of age, sex and dementia diagnosis (Table 1).

Of the 276 residents or third parties who declined participation in the SIMPLER study, reasons were recorded for 84 residents and included lack of interest, concern about potential undue resident distress, and perceived lack of benefit (Box 2). Discussions with residents suggested that altruism was an important factor underpinning resident participation.

“*Give back for the future and for other residents… if you want better conditions for yourself, you surely want them for other people too*.”

[Resident 1]

“*I think my main thought there is how it’s going to benefit all of us*.”

[Resident 3]

Other factors included communication and promotion, relationships and trust, and willingness to change the current medication regimen.

Box 2 **Most frequent reasons provided when the invitation to participate in the SIMPLER study was declined by the resident or the third-party.**

- The resident, or the family, are not interested in participating in the study (*n* = 19)
- The resident is too confused or distressed, or the third-party does not wish to confuse, distress or both the resident (*n* = 13)
- The study or intervention is perceived as unnecessary, inappropriate, or would not benefit the resident (i.e., because the resident does not take many medications) (*n* = 12)
- The resident has been unwell recently (*n* = 8)
- The resident, or the third-party, are busy or lack time needed for involvement (*n* = 8)
- The resident has recently had their medications checked, or changed, whereby they do not want any further changes (*n* = 5)
- It is perceived that the resident would not be able to participate in the study, or would not be a beneficial participant (*n* = 4)

#### 3.2.1. Communication and Promotion 

Residents appreciated the research nurses taking the time to explain the study on an individual basis. This process relied on resident availability and research nurses reported difficulty in finding mutually convenient times to discuss with third parties. A personal amplifier was used for recruitment discussions with residents with hearing impairment if required. The information and consent forms were designed with consumer input. However, the complexity of the 3-page information sheet, while necessary to inform the decision to participate, was a perceived barrier to recruitment. Residents with visual impairment were provided with large font information sheets as needed and in a small number of cases, materials were read aloud to residents. 

Delays with third parties receiving materials due to postal delivery times made recruitment discussions more difficult and delayed obtaining written consent. Research nurses perceived face-to-face discussions as more effective during recruitment.

“*We were seeing that we were getting greater acceptance of the project through contact face to face than what we were over the phone*.”

[Research Nurse 1]

“*It was only when we were first on the phone contacting the next of kin that we found that barrier was there. Not when I was actually on site talking to them*.”

[Research Nurse 3]

An open discussion of the research at resident events and in newsletters facilitated recruitment. The perceived impact of posters in the RACF was limited due to the large number of other posters. Recruitment was further facilitated through drawing upon altruism, an individual’s appreciation of the importance of research within society, benefits and relevance to older people, and direct personal benefits. Research nurses emphasized access to a new service at no charge and the opportunity for a ‘quality check’ from an independent pharmacist. Presentation of a case study highlighting resident and staff benefits was suggested to facilitate recruitment in future studies.

#### 3.2.2. Relationships and Trust 

The opinion of trusted persons (i.e., family, GP, RACF staff) influenced participation decisions. Recruitment barriers included resident desire to avoid offending their GP, particularly through involvement of a pharmacist not involved in their usual care.

“*They [the residents] felt that they didn’t want to offend their GP by having somebody else look at things that the GP was already in control of*.”

[Research Nurse 1]

“*It’s almost like not wanting to offend the doctor or just trusting that the doctor’s got it all right.*”

[Research Nurse 3]

Emphasizing the interdisciplinary approach was perceived to facilitate residents’ willingness to participate. An introduction to the resident by a staff member involved in their usual care increased trust and improved recruitment.

“*I think it was easier being introduced by a care worker or nursing staff when you go in the room. I know it sounds a bit silly but it’s almost like you’re not intruding… But we couldn’t always do that because the staff were busy*.”

[Research Nurse 3]

One research nurse perceived that recruitment was facilitated by having a family member present during an initial discussion to aid resident comfort and trust. Another research nurse suggested holding information sessions on weekends when more family members are visiting the RACFs.

#### 3.2.3. Resistance to Change the Current Medication Regimen

Some residents were concerned about changing their medication regimen for fear of losing control or knowledge of medication-taking patterns.

“*…some people have been, you know, taking their tablets in a certain way for a long time prior to them coming into residential care and…they don’t like change and they’re very reluctant and concerned that it’s going to affect their health… Or they’ve had previous bad experiences of changing medications*.”

[Registered Nurse 4]

“*They had that thought that their medication was going to be changed to something different than what they were already taking, so there was a bit of explaining… that it wasn’t actually going to change what they were taking*.”

[Recruitment Assistant 1]

To address these concerns, research nurses explained that the study identified opportunities to simplify rather than change or discontinue medications and emphasized the involvement of the resident and health care professionals in implementing recommendations.

### 3.3. Delivery to RACFs and Individual Residents

The clinical pharmacist was trained on the use of MRS GRACE and intervention delivery by two researchers (JKS, EYH) prior to intervention commencement. The median time from resident recruitment to intervention delivery was 28 days (Table 2) because the pharmacist waited for a group of residents to be recruited before travelling to each RACF. Three residents died pre-intervention for reasons unrelated to study involvement.

The clinical pharmacist felt positive about intervention delivery. Medication charts were examined for all participants and care was taken to minimize the time the charts were unavailable to staff. Nursing notes were only referred to when needed to determine an indication for a specific medication.

“*There were no issues around it [intervention delivery]. They [the clinical pharmacist] worked around when staff were doing their medication round and yeah it seemed to all go fairly quickly and smoothly, I can remember*.”

[Senior Registered Nurse 4]

Inspection of the clinical pharmacist’s records, together with the descriptions provided by interviewees, showed intervention delivery was generally consistent with the study protocol despite the pharmacist not referring to MRS GRACE each time. This was because the principles outlined in MRS GRACE had become embedded as part of the pharmacist’s practice, which was consistent with how the tool would likely be used future routine practice. The first step of MRS GRACE is to discuss medication simplification with the resident; however, to maximize efficiency of intervention delivery, the pharmacist determined if simplification was possible (steps 2 to 5 of MRS GRACE) prior to attempting a discussion with a resident for whom simplification was possible. Discussions with residents and/or family members were considered an important component of the intervention but were time consuming to organize and other medication-related issues were sometimes mentioned by residents.

“*Talking with residents, well in the project I guess there was, talking with residents, they don’t necessarily understand that you’re just there as a project pharmacist, and they want you to change all sorts of medicines and things and I had to explain no, it was just around the simplification and that they’d need to see their regular pharmacist around other issues*.”

[Clinical Pharmacist]

Resident discussions could not be undertaken when a resident was not present or was unwell. When a resident discussion could not be undertaken, the pharmacist brought this to the attention of the senior RN and GP in the report and had a face-to-face discussion with an RN about the suitability of the simplification recommendation. The pharmacist attempted to call the resident’s GP when simplification was possible and often left messages with GP practice staff. The clinical pharmacist prepared a report with recommendations using a structured template (available on request). Reports were not prepared when simplification was not possible. Intervention delivery was recorded on the medication administration chart regardless of whether simplification was recommended.

Table 2 describes intervention delivery times; the pharmacist advised that intervention delivery would be quicker during routine clinical practice. The pharmacist debriefed with senior RNs at the end of each day to discuss recommendations, and later discussed the site visit with the embedded researcher (JKS). The pharmacist suggested a follow-up Residential Medication Management Review (RMMR; an Australian Government-funded comprehensive medication review) for four residents but anecdotally reported that multiple residents could benefit from an RMMR.

### 3.4. Response of RACFs

Following the pharmacist’s visit, changes to medication administration times required approval and implementation by the RACF manager, senior RN, or GP, while other simplification recommendations (e.g., switching to long-acting/combination products) could only be implemented by the GP. Senior RNs were the main drivers of implementation and utilized strategies such as collating the pharmacist reports and setting aside time to implement recommendations in bulk, integrating the report review into their usual workload when providing care for that specific resident, delegating to the RN perceived to have the best knowledge of that resident, and/or liaising with the GP. Good working relationships between RACF staff and GPs, and an organizational culture that valued medication safety were perceived facilitators for implementation. 

The clinical pharmacist perceived some variation existed between RACFs in the types of simplification recommendations made and the facility response to the intervention.

“*And different sites had different issues. Like one site, they’d already virtually simplified all the times themselves, I think that was the last site that I went to. So, it was really only doctor interventions. Whereas other sites had lots of timing interventions and not many doctor interventions*.”

[Clinical Pharmacist]

“*Yeah, and I think that’s what you’d expect from human nature everywhere, that they’ve got a number of priorities in the facility and so depending on, yeah, what their other competing priorities were, some sites were super keen, other nurses I don’t, they just saw it as an extra workload*.”

[Clinical Pharmacist]

This was also demonstrated at 4-month follow-up, where intervention implementation rates at the four RACFs were 25%, 59%, 62% and 86%. Implementation of recommendations was aided by the aged care provider’s multidisciplinary Medication Advisory Committee (MAC) agreeing on a protocol for implementation of the pharmacist’s recommendations prior to SIMPLER commencement. The RACF with the lowest implementation rate had postponed study commencement to accommodate competing quality improvement commitments, had the lowest recruitment rate and the highest proportion of recommendations that could only be implemented by GPs (i.e., not by senior RNs). The RACF with the highest recruitment rate also had the highest implementation rate. In this RACF, the senior RN championed the project through integrating simplification into their routine work and fostering relationships with local GPs and pharmacists. This RN described a participant with improved quality of life following simplification of analgesia, and later transferred the learnings when providing care for other residents in a similar situation.

“*And because we had that knowledge and you know we actually had another resident with a similar thing and we said look last time they said, this pharmacist said we could use a slow-release morphine tablet. It was just as effective and so you know we were able to use the knowledge that we got through the study for somebody else*.”

[Clinical nurse 4]

At another intervention RACF (with the second lowest recruitment rate), the senior RN reviewed the simplification recommendations but requested support with implementation due to other commitments. It was suggested that implementation could be improved by sharing of examples of how it can be incorporated into normal workflows.

Other RACF staff generally responded positively to intervention delivery and implementation. Occasional negative responses included misconception that the study would possibly lead to reduced working hours for nurses, and the perception that the pharmacist’s recommendations were minor.

“*I just think that some staff are more positive about research than others, basically. But in saying that I don’t think there was... I think all of the sites, there were some staff at the sites that were very positive about it but there was also some that were not so positive. But they were a minority*.”

[Research nurse 2]

Sometimes RNs were hesitant to implement recommendations to change an administration time without consulting the GP. Effective communication and working relationships aided implementation. It was also felt that implementation was aided by having the pharmacist onsite at the same time as the GP. The research team had integrated SIMPLER into the existing organization-wide quality improvement program and senior staff at several RACFs reported using these materials during facility audits that assess compliance with national RACF quality standards.

Post-intervention, initial outcomes met expectations for some nurses, although others expected a greater impact. Some nurses described a reduction in time spent administering medications at specific times (e.g., reduced administrations during the 2 pm round was perceived as beneficial due to difficulties locating residents participating in social activities).

“*It just saves everyone time and doesn’t affect quality of the service, which is important… Definitely less for me to do during lunch run, which used to be busier … And I mean, I don’t spend an extra time in the morning, because if I give one extra, two extra tablets in the morning, that doesn’t make difference in the time I spend with the residents… But yeah, probably it benefits residents because I have more time to be involved in care planning or writing progress notes, which are always good to document…*”

[Enrolled Nurse 1]

Other nursing staff perceived nil or insignificant impact on workload during medication administration rounds. One nurse suggested that future interventions could instead focus on eliminating an entire administration round for all residents at an RACF where possible.

### 3.5. Response of Residents and Other Individuals

Residents and family were generally positive about the concept of medication simplification and trial participation despite varied recall of the intervention among the residents interviewed.

“*I think having another set of eyes coming through is good... Because she is, she’s on a hell of a lot of medication and if they’ve reduced the number of times that they’re giving to her, and they’re sort of giving it to her in one go, at least it’s not as disruptive*.”

[Resident family representative 1]

However, RACF staff, residents and family members perceived varied benefit in terms of resident quality of life. The clinical pharmacist described not recommending simplification for a resident who appeared anxious about potential regimen changes. Another resident withdrew after the intervention for reasons unrelated to the study.

The GPs and community pharmacists also responded positively and considered simplification to be low risk if recommendations were individualized.

“*I guess I probably don’t pay too much attention to the times the medications are given and maybe I should do that more. But yeah, I mean, because I think the two that I got, it was just like, oh yeah, well that makes sense. You know, let’s do that. I hadn’t really noticed that that was an issue. Yeah, which is probably good. Probably means that there’s good reason to do this sort of thing*.”

[General Practitioner 1]

“*I did get some response back from two of the GPs saying yep, absolutely, really, really happy to support this*.”

[Research Nurse 1]

“*So yeah, it was one of those things that I thought this can only help and benefit everybody*.”

[Community Pharmacist 2]

The GP perceived the simplification recommendations as easy to understand and implement. Both community pharmacists perceived the amount of time required to make changes to medications packed in the resident’s dose administration aids post-simplification was negligible, and viewed the study as supporting an expanded role for pharmacists to deliver clinical services to RACFs.

### 3.6. Future Implementation

Most of the health professionals interviewed supported broader implementation, with only minor modifications suggested. Pharmacists were viewed as having the comprehensive pharmacological knowledge required to identify medication simplification opportunities. Most interview participants agreed medication simplification should be undertaken when a resident first enters the RACF. Two approaches were suggested regarding delivery thereafter. The ‘routine approach’ proposed regular delivery to all residents every 4–12 months, whereas the ‘needs approach’ focused on targeting residents with multiple administration times, recent changes to medications/health status and medication refusals, or at the discretion of the RACF nurses, pharmacist, or GP. Strategies suggested to identify residents who may benefit included screening tools and clinical champions.

“*So, I think at point of entry would be a really, really good start. And then perhaps at, you know, significant health changes. I mean, could it be done annually and a medication review as well*?”

[Research Nurse 1]

“*I don’t think you have to be automatic process, you know, it’s just on the needs of every [resident], needs are different. So definitely admission, definitely significant health status change, hospitalization. And then probably do some regular ones, but not too often. And then again, a RN or CN can make that call as well, to inform. So that’s one part of getting nurses involved, because they can easily, they can make that call and inform pharmacist.*”

[Enrolled nurse 1]

“*So, if I was, ideally, in an ideal world, if I was able to commit as much time as I like to a facility and could be there all the time, I’d like to do it once a quarter as a routine thing and for all residents that came back from hospital.*”

[Clinical pharmacist 1]

Good communication skills and interdisciplinary collaboration were perceived as facilitating implementation. The ability to build rapport with residents and family members, familiarization with RACF workflow, consistent working hours, and a streamlined approach to communicating recommendations were suggested to support service delivery. It was suggested that implementation could be further facilitated by raising public awareness about the potential for simplification and role of the pharmacist. 

There were mixed opinions on how best to integrate a medication simplification service with existing medication services provided in Australian RACFs. Some participants viewed medication simplification as a stand-alone service, while others suggested it could be incorporated into existing government-subsidized medication management services. Barriers to integration into existing models of care included competing clinical activities. Participants desired government funding to implement this new service although some suggested that aged care provider organizations could fund the service. The time required for RACF nurses and GPs to implement recommendations was perceived as a potential barrier to wider implementation, while others perceived it could be offset by possible reductions in the time required to administer medications.

### 3.7. Unintended Consequences

The organization’s research and development team appreciated the upskilling of staff in research methods and subsequent implementation of new research systems. Residents appreciated the indirect benefits of interacting with research nurses. Compliments and critical feedback unrelated to SIMPLER were fed back to senior RNs. The research nurses described building rapport with RACF staff and alerted senior RNs to problems with medication charts and when residents were perceived to be at risk of poor outcomes (e.g., nutritional assessment suggested possible malnutrition). RACF staff were exposed to new clinical assessments through their participation as staff informants. Some expressed an interest in using these assessments in the future, while others were interested to learn about research. One research nurse described showcasing research and career development opportunities to RACF staff to encourage their continuing professional development.

While providing the intervention, the clinical pharmacist noted opportunities to improve delivery of RMMRs at one RACF, prompting an organizational review of RMMR delivery. Interview participants appreciated the increased educational opportunities for staff during the pharmacist’s visit. The intervention prompted a local pharmacist and nurses to subsequently implement medication simplification while conducting RMMRs at two other RACFs.

## 4. Discussion

This process evaluation found that SIMPLER was generally implemented as planned. Despite facility variation, a representative resident sample was recruited. The intervention was delivered consistently; however, implementation of the clinical pharmacist’s recommendations varied considerably between the RACFs and staff relationships with local GPs and community pharmacists were an anecdotal predictor of implementation. Overall, the study was viewed positively, with few negative experiences reported. Stakeholders also identified opportunities to disseminate simplification more widely.

Clinical trials in RACFs are resource intensive, and effective recruitment strategies are paramount to avoid resource wastage and insufficient sample sizes [14,28]. The 38.4% SIMPLER participation rate is similar to several other studies in RACFs [4,12,15,29]. Recruitment rates were slightly higher among residents for whom third party consent was required compared to those who were able to provide consent to participate (41.8% versus 32.6%). Reasons provided for non-participation (e.g., concern about potential undue resident distress or burden, recent illness, lack of time and perceived lack of benefit) were similar to those encountered by others when recruiting people living in RACFs, people with dementia or third parties to participate in trials [12,30]. Despite these factors and other known challenges in recruiting residents with cognitive impairment to participate in trials [31,32], SIMPLER participants were representative of all residents in terms of age, sex and dementia diagnoses. Study promotional activities helped to raise awareness and face-to-face recruitment discussions were preferred. This is consistent with existing literature that suggests considerable planning and direct methods of communication can aid recruitment [14,32]. Lack of familiarity with research in some RACFs, concurrent quality improvement activities, and differing levels of health literacy were perceived to contribute to variation in recruitment. Using pilot-tested and easily understandable materials that incorporate a mix of plain language explanations and visuals, and research nurse training in interactive techniques can aid consumer decision-making about research participation [33]. However, the intervention RACFs with the highest recruitment rates also had the highest intervention implementation rates, suggesting RACF organizational culture and appreciation for the study objectives/benefits were also important facilitators. We suggest emphasizing the following during recruitment discussions: the interdisciplinary project nature and shared decision-making; delivery by experienced health professionals; ability to withdraw at any time; that all residents can benefit, including those who are frail or experiencing cognitive impairment; how trial processes can be adapted for residents who are not able to self-report information; and drawing on altruism. Additional strategies mentioned in the literature include mailing study postcards/short flyers to third parties prior to recruitment, offering financial incentives, using concise information sheets, and emphasizing the presence of the trial monitoring committee [12,14,15,22].

The intervention was delivered to all but three residents in the intervention arm. No major concerns with the quality of delivery were encountered. Intervention fidelity was supported by utilizing an experienced clinical pharmacist who was trained in the use of a validated simplification tool. The proportion of residents for whom medication simplification was possible was consistent with two previous studies [9,22]. Resident or third-party discussions are key to understanding resident preferences [9] and explaining the likely impact of simplification on specific medication administration times and formulations prescribed. However, the pharmacist reported difficulty arranging resident/family discussions and the outcomes of subsequent RN/GP discussions with the resident or family are unknown. Future studies should incorporate additional resources and strategies to support best-practice shared decision making in medication management interventions in RACFs [34]. Evidence from a process evaluation of a cluster RCT of a multifaceted primary care intervention suggests that potentially inappropriate medications may be more likely to be withdrawn when patients are present during a GP’s review of their medication regimen [19].

At 4-month follow-up of SIMPLER, at least one of the pharmacist’s simplification recommendations had been implemented in 46 residents (74%), demonstrating the acceptability of the intervention [3]. However, there was considerable variation in responsiveness between facilities and implementation prompts from the research team were required for some RACFs. Similar to a cluster RCT of an intervention to enhance medication use in Belgian RACFs [17], key drivers of implementation included good stakeholder working relationships, presence of a clinical champion, ability to integrate into normal workflows and appreciation for the potential benefits of medication simplification. Although few negative responses or unintended consequences were reported, the perceived impact of the intervention for residents and staff varied. Implementation uptake could be enhanced through wider engagement with GPs prior to the intervention, engaging the clinical pharmacist to formally follow-up with senior RNs and GPs post-intervention, repeated reinforcement of the potential benefits of simplification suggested by stakeholders (e.g., reduced interruptions for residents, reduced time-related pressure on staff decision-making, and more time for staff to devote to resident interactions, clinical documentation, handover, and provision of non-pharmacological interventions for residents with dementia) and via resident and family discussions, to further enhance the reduction in the number of medication administration times observed in the intervention arm of the SIMPLER study (i.e., the primary outcome) during follow-up [3,6].

The SIMPLER intervention was delivered as a stand-alone clinical service. Participants identified opportunities for integrating medication simplification into routine practice, suggesting that simplification could be incorporated into a pre-admission ‘package’ of activities that a pharmacist provides on RACF admission and thereafter, either when clinical circumstances change or at routine intervals. Transitions of care are considered a risky period for medication safety due to changes in medication use and healthcare providers [35,36], and medication reconciliation is recommended before undertaking simplification [9]; hence, provision at RACF entry is a timely opportunity for intervention. One participant suggested that prescribers could use MRS GRACE to guide simplification when preparing new medication charts as a potential point for future intervention. Previous studies in other settings have utilized screening tools, algorithms and/or electronic medication chart prompts to identify individuals with complex medication regimens [37,38,39]. Additionally, in the Australian state of Victoria, a medication quality indicator ‘percentage of residents with more than four medication administration times’ was developed, validated and implemented across all 180 public-sector RACFs as a prompt to reduce unnecessary complexity [16]. Similar approaches could be considered to identify residents who may benefit from simplification. The sustained reduction in the number of medication administration times at 8- and 12-month follow-up of the SIMPLER study [6], observed after the process evaluation was conducted, provides further support for medication simplification to be delivered at least annually.

Features of other successful RACF interventions, such as clinical champions, interdisciplinary collaboration, and good working relationships [16,19,40,41] were identified as facilitators to wider implementation. Remuneration for stakeholders was also considered important, which is consistent with findings of a process evaluation of medication intervention trial in Belgium RACFs [17]. Undertaking an economic evaluation that considers the benefits and costs of the SIMPLER intervention may also facilitate wider uptake. Employment of pharmacists to deliver clinical and educational services, and contribute to clinical governance activities in RACFs is an area of increasing focus in Australia [42,43]. The recent Australian Royal Commission into Aged Care Quality and Safety recommended regular RMMR provision for residents, and for pharmacists to be engaged by aged care provider organizations to provide clinical services [44]. However, in situations where clinical pharmacy services are not routinely provided, stakeholders suggested the simplification intervention could be incorporated into the following existing RACF-based services: (i) a RMMR (an Australian Government-funded comprehensive medication review for residents of RACFs) that is delivered to an individual resident or (ii) a Quality Use of Medicines activity which provides facility-level support (e.g., educational or continuous quality improvement initiatives) to enhance medicines use [16]. Pharmacists integrating simplification activities into their usual practice in RACFs would already have established relationships with stakeholders, which could address some of the recruitment and implementation barriers encountered in SIMPLER. Additionally, recent RMMR program changes that enable pharmacists to provide up to two follow-up visits [35] would enable pharmacists to follow-up on simplification recommendations made during an RMMR and prompt implementation if appropriate.

### Strengths and Limitations

This mixed-methods process evaluation was guided by a framework for cluster RCTs [11] and explored an area of need. Comprehensive SIMPLER recruitment logs enabled us to extract information about why informed consent was not provided for some, but not all residents. Hence, we were unable to determine relationships between specific resident-related factors that may have made it difficult to participate in research (e.g., frailty, independence with activities of daily living [30]) and participation in SIMPLER. We did not collect information on the reasons why third-party contact was required during recruitment. Additionally, the retrospective nature of the document analysis meant the total number of attempts made by research nurses to have a face-to-face resident recruitment discussion is likely underestimated. The time spent delivering the intervention by the single pharmacist may not be representative of all pharmacists. We suggest future studies collect information on time spent on other activities (e.g., organizing resident/family discussions, implementing recommendations) to guide future implementation and remuneration decisions.

Importantly, views from most stakeholder groups were captured, except for residents who declined to participate in SIMPLER. Only one GP could be interviewed, and their views may not be representative of other GPs, particularly those who chose not to implement recommendations. We did not seek input from allied health professionals on the impact of the intervention. Interview questions were piloted beforehand, however as both interviewers had a pharmacy background this could have biased discussions towards pharmacist-related priorities. Implementation fidelity was assessed through pharmacist self-report and document analysis, however prospective measures (e.g., repeated pharmacist training, checklists, and direct observation and feedback [45]) are recommended in future studies.

## 5. Conclusions

This process evaluation found the SIMPLER study was generally delivered as planned and in accordance with the study protocol. All of the RACFs approached agreed to participate. Although recruitment was resource intensive, a representative sample of residents was recruited. No major issues were found with the trial processes or implementation of the intervention, and any unintended consequences were largely perceived as beneficial. Stakeholders were supportive of wider implementation of medication simplification. Opportunities exist to integrate medication simplification into routine practice, with only minor modifications suggested by stakeholders. 

The barriers and enablers identified in this process evaluation can inform future interventions to optimize medication management in RACFs. We suggest ensuring sufficient time and resources are allocated for face-to-face recruitment discussions, continual promotional activities, and utilizing recruitment strategies that draw on altruism, build trust with potential participants and address concerns about potential changes to medications. Overall findings suggest that an appreciation for the potential benefit of the intervention, effective communication between stakeholders (including residents and family members) and good working relationships are vital for successful implementation of medication management interventions in RACFs.

## Figures and Tables

**Table 1:** Characteristics of the Australian residential aged care population, all permanent residents of RACFs operated by the aged care provider organization, and residents participating in the SIMPLER study.

Characteristics	Australian Permanent RACF Population ^a^ (*n* = 178,713 Residents)	Permanent Resident Population within the Participating Organization (*n* = 703, 8 RACFs) ^c^	Residents Participating in SIMPLER		
			All Residents (*n* = 242, 8 RACFs)	Residents at Intervention RACFs (*n* = 99, 4 RACFs)	Residents at Comparison RACFs (*n* = 143, 4 RACFs)
Age (years), mean (SD)	59% aged ≥ 85 years ^a^	86.4	86.0 (8.1)	85.7 (7.8)	86.2 (8.3)
Male (*n*, %)	58,104 (32.5%) ^a^	180 (25.6%)	63 (26.0%)	32 (32.3)	31 (21.7%)
Dementia (*n*, %)	52% ^b^	356 (50.6%)	131 (54.1%)	54 (54.6%)	77 (53.9%)
Metropolitan location (*n*, %)	124,348 (69.6%) ^a^	529 (75.2%)	194 (80.2%)	67 (67.7%)	127 (88.8%)

^a^ as at 30 June 2017 [26]; ^b^ as at 20 June 2016 [27]; ^c^ as at 7 July 2017.

**Table 2:** Overview of intervention delivery by the clinical pharmacist (*n* = 96 residents).

	N (%) or Median (IQR)
Time between study entry and intervention delivery (days)	28 (15–35)
Total number of days spent delivering the intervention at each RACF [range]	1 to 3
Pharmacist was able to discuss simplification recommendations with resident and/or family ^a^	20 (31.7%)
Number of residents or family members who did not want to proceed with simplification when it was possible ^b^	1 (5.0%)
Time spent delivering intervention (minutes)	
Generating recommendations	12 (12–12)
Report preparation & communicating findings to RN/GP ^c^	20 (20–20)
Speaking with residents and/or family ^b^	15 (15–15)
Total time spent per resident	32 (12–35)
No. of residents for whom the pharmacist recommended a referral be made for a Residential Medication Management Review	4 (4.1%)

^a^ of the *n* = 63 residents for whom simplification was possible, ^b^ among *n* = 20 residents for whom simplification was discussed with resident/family, ^c^ for *n* = 62 residents for whom ≥1 medication simplification recommendation was made by the clinical pharmacist.

## Data Availability

Supporting data are not available as study participants did not agree for their data to be shared publicly.

## Supplementary Materials

The following are available online at https://www.mdpi.com/article/10.3390/ijerph18115778/s1, Table S1. Overview of the methods and 4-month outcomes of the SIMPLER cluster randomized controlled trial [1,3,6,7,8,9], Table S2. Summary of research questions, methods, and data sources for the process evaluation, adapted from Grant et al. [11].
