# Supplementary material for: Process Evaluation of the SImplification of Medications Prescribed to Long-tErm Care Residents (SIMPLER) Cluster Randomized Controlled Trial: A Mixed Methods Study
Source: Int J Environ Res Public Health. 2021 May 27;18(11):5778. doi: 10.3390/ijerph18115778 (PMC8199013; doi:10.3390/ijerph18115778)
Supplement: Supplementary file 1 [file ijerph-18-05778-s001.zip › ijerph-1200012-supplementary.pdf]

Supplementary Table S1. Overview of the methods and 4-month outcomes of the SIMPLER cluster randomized controlled trial [1, 3, 6-9].

Methods	Overview
Study aim	To investigate the application of a structured process to consolidate the number of medication administration times for individuals accessing residential aged care services
Participants	242 permanent, English-speaking residents (intervention n=99, comparison arm n=143) with a median age of 87 years (IQR 81-92) were recruited from eight RACFs located in South Australia between April and October 2017. At study entry, the median Charlson Comorbidity Index score was 2 (IQR 2-3), the median Katz Index of Independence in Activities of Daily Living score was 1 (IQR 1-3) and the median Frailty in Nursing Homes (FRAIL-NH) score was 7 (IQR 3-10). At study entry, participants were charted medications for regular administration an average of four times daily.
Intervention group	Delivery of the intervention involved an experienced clinical pharmacist visiting the RACF to apply the Medication Regimen Simplification Guide for Residential Aged CarE (MRS GRACE) tool to identify opportunities to simplify medication regimen for the participating residents. The pharmacist identified opportunities to simplify the medication regimen for a participating resident and communicated these recommendations to senior RNs and the resident's GP.<br>MRS GRACE is an implicit tool designed to guide the process of medication simplification in RACFs. The tool, which was validated by two pharmacists, encourages the user to work through the following five key questions when determining whether a medication regimen can be simplified:<br>1. Is there a resident related factor that precludes simplification?<br>2. Is there a regulatory or safety imperative that precludes simplification?<br>3. Is simplification likely to result in any clinically significant drug–drug, drug–food, or drug–time interactions?<br>4. Is there no alternative formulation available that can support less complex dosing?<br>5. Is simplification likely to result in any unintended consequences for the resident or facility?<br>Further information about the development and validation of MRS GRACE, and the accompanying explanatory statement, is available from the following open access publication: [9]
Comparison arm	Residents who participated in the study from the 4 comparison RACFs received usual care.
Outcome measures	• The primary outcome was the number of medication administration times per day for medications charted regularly at 4-month follow-up.<br>• Secondary outcomes were resident satisfaction and quality of life.<br>• Harms were falls, medication incidents, hospitalizations, mortality.
Main findings	• Medication simplification was possible for 62 (65%) of the 96 residents who received the intervention. Recommendations often focused on changing the time of medication administration (65%). Just over half (58%) of all simplification recommendations could potentially be implemented by a senior RN at the RACF.<br>• At 4-month follow-up, 57 (62%) of the 92 medication simplification recommendations had been implemented. Among the 62 residents for whom simplification was possible, at least one simplification recommendation was implemented at 4-month follow-up in 46 (74%) residents.<br>• The mean number of medication administration times at 4-month follow-up was reduced in the intervention arm in comparison to usual care (-0.36, 95% CI -0.63 to -0.09, p=0.01).<br>• No difference in resident satisfaction or quality of life at 4-month follow-up between the study arms was observed.<br>• No significant differences in harms were observed between the study arms at 4-month follow-up.

CI confidence interval, GP general medical practitioner, IQR interquartile range, MRS GRACE Medication Regimen Simplification Guide for Residential Aged CarE, RACFs residential aged care facilities, RCT randomized controlled trial, RN registered nurse, SD standard deviation, SIMPLER SImplification of Medications Prescribed to Long-tErm care Residents.

Supplementary Table S2. Summary of research questions, methods, and data sources for the process evaluation, adapted from Grant et al. [11].

Domain	Research question	Research methods and/or data sources[a]
Recruitment of RACFs	How were the RACFs sampled and recruited?<br>Who agrees to participate?<br>Why do the RACFs agree to participate (or not)?	• Document analysis of recruitment logs.<br>• Semi-structured interviews with the aged care provider staff and research team.
Recruitment and reach among residents	Who receives the intervention in each setting?<br>Are they representative?<br>What factors impacted on resident recruitment?	• Document analysis of clinical pharmacist logs and intervention reports prepared by the pharmacist.<br>• Semi structured interviews with all stakeholders.<br>• Quantitative analysis of baseline data collected for the SIMPLER study, data provided by the aged care organization and publicly available data.
Delivery to RACFs	What intervention is delivered for each RACF?<br>Is it the one intended by the researchers?	• Document analysis of clinical pharmacist logs and intervention reports (quantitative and qualitative).<br>• Semi-structured interviews with clinical pharmacist and aged care provider staff.
Delivery to individual residents	What intervention is delivered to the resident?<br>Is the delivered intervention the one intended by the researchers?	• Document analysis of clinical pharmacist logs and intervention reports (quantitative and qualitative).<br>• Semi structured interviews with all stakeholders.
Response of RACFs	How is the intervention adopted and supported by individual RACFs?	• Document analysis of research team records (quantitative and qualitative).<br>• Semi-structured interviews with all stakeholders.
Response of residents and other individuals	How does the target population respond?	• Document analysis of clinical pharmacist's logs.<br>• Semi-structured interviews with all stakeholders.
Future implementation	What is the feasibility, facilitators, and barriers to wider implementation of the intervention?	• Semi-structured interviews with all stakeholders.
Unintended consequences	Are there any unintended changes in processes and outcomes, both related to the trial intervention and unrelated care?	• Document analysis of records maintained by the research team and the clinical pharmacist.<br>• Semi-structured interviews with all stakeholders.
Theory	What theory has been used to develop the intervention?	• Principles of the theoretical framework underpinning Australia's National Strategy for Quality Use of Medicines [23] and existing medication management activities in Australian RACFs [16] were considered in the design of the intervention and when interpreting process evaluation findings.
Context	What is the wider context in which the trial is being conducted?	The SIMPLER study setting and context are outlined in the introduction and methods section, and in Supplementary Table 1.

[a] Data were utilized in the qualitative analysis unless stated otherwise. RACF residential aged care facility, SIMPLER SImplification of Medications Prescribed to Long-tErm care Residents.
